# Warnings in early narrative assessment that might predict performance in residency: signal from an internal medicine residency program

**DOI:** 10.1007/s40037-021-00681-w

**Published:** 2021-09-02

**Authors:** Matthew Kelleher, Benjamin Kinnear, Dana R. Sall, Danielle E. Weber, Bailey DeCoursey, Jennifer Nelson, Melissa Klein, Eric J. Warm, Daniel J. Schumacher

**Affiliations:** 1 Department of Pediatrics, University of Cincinnati College of Medicine, Cincinnati, OH USA; 2 HonorHealth Internal Medicine Residency Program, Scottsdale, Arizona and University of Arizona College of Medicine, Phoenix, AZ USA; 3 Department of Internal Medicine, University of Cincinnati College of Medicine, Cincinnati, OH USA

**Keywords:** Assessment, Narrative data, Competency-based medical education, Competency committees, Qualitative research

## Abstract

**Introduction:**

Narrative assessment data are valuable in understanding struggles in resident performance. However, it remains unknown which themes in narrative data that occur early in training may indicate a higher likelihood of struggles later in training, allowing programs to intervene sooner.

**Methods:**

Using learning analytics, we identified 26 internal medicine residents in three cohorts that were below expected entrustment during training. We compiled all narrative data in the first 6 months of training for these residents as well as 13 typically performing residents for comparison. Narrative data were blinded for all 39 residents during initial phases of an inductive thematic analysis for initial coding.

**Results:**

Many similarities were identified between the two cohorts. Codes that differed between typical and lower entrusted residents were grouped into two types of themes: three explicit/manifest and three implicit/latent with six total themes. The explicit/manifest themes focused on specific aspects of resident performance with assessors describing 1) Gaps in attention to detail, 2) Communication deficits with patients, and 3) Difficulty recognizing the “big picture” in patient care. Three implicit/latent themes, focused on how narrative data were written, were also identified: 1) Feedback described as a deficiency rather than an opportunity to improve, 2) Normative comparisons to identify a resident as being behind their peers, and 3) Warning of possible risk to patient care.

**Discussion:**

Clinical competency committees (CCCs) usually rely on accumulated data and trends. Using the themes in this paper while reviewing narrative comments may help CCCs with earlier recognition and better allocation of resources to support residents’ development.

## Introduction

Competency-based medical education requires assessment of performance in authentic clinical learning environments via workplace-based assessments (WPBAs) [1–3]. Many WPBAs use a combination of quantitative performance ratings and narrative comments [4]. Historically, educators have viewed quantitative assessment data as more reliable and useful for summative decision-making, and narrative data as subjective and useful for formative feedback [5]. However, quantitative WPBA practices can result in psychometrically poor results,[6] leading some to advocate for a “post-psychometric” era of assessment in which the subjective and contextual natures of WPBAs are embraced [7–9]. This has sparked interest in narrative data as a potentially more useful measure of performance than quantitative assessment data [5, 10–13].

Estimates suggest anywhere from 5 to 10% of residents “struggle” during their training, with many problems identified too late to maximize help to the learner [14–17]. Multiple studies have shown the value of narrative data in making summative decisions, while others have attempted to predict ongoing struggles with learner development [12, 18–20]. However, challenges exist when using narrative data in these ways. Assessors frequently “code” their comments, or use nonspecific and idiosyncratic language, requiring readers to interpret hidden meaning [21–24]. Patterns in narrative data often take time to develop, with themes only becoming clear after months or years of training. Comments can also be discordant from one another or from accompanying quantitative data, making interpretation and subsequent decision-making challenging. Narrative data are also cumbersome and time-consuming to analyze, particularly in large training programs where many comments are present [25]. Because of these challenges, training programs may struggle to use their narrative data to their full potential, particularly with residents early in training.

Carefully collected and analyzed narrative assessment data can provide value, particularly early in training given their potential to capture rich details about a learner’s performance. Different approaches to finding value in narrative data have included the use of “keyword algorithms” or counting the number of words and percentage of assessments containing negative or ambiguous comments, which were associated with the need for remediation [15, 19]. However, using keyword algorithms was much better at ruling out, rather than predicting who would struggle as evidenced by a positive predictive value of 23% [19]. Using negative or ambiguous comments was helpful when reading an entire residency file, but 12 of 17 residents in good standing still had negative comments [15]. These approaches highlight opportunities to better understand how narrative feedback analyzed early in training may differ between residents who struggle from those who do not struggle, while also describing the actual differences in the content of narrative data. In this study, we aimed to explore the first 6 months of narrative comments from WPBAs in one internal medicine residency program, with a robust program of assessment,[26–29] to determine identifiable patterns that subsequently might predict who will receive lower quantitative entrustment ratings over the course of training. Recognizing the early signal that portends the need for additional support and intervention at the beginning of residency can provide a practical approach for clinical competency committees (CCCs) to surface what matters from the plethora of positive, nonspecific, and or idiosyncratic narrative feedback.

## Methods

### Setting

This study was conducted at the internal medicine residency program at the University of Cincinnati College of Medicine. In this medium-sized program (approximately 89 categorical residents across 3 years of training), faculty members, peers, and allied health professionals (AHPs) such as nurses, pharmacists, and therapists assess residents using discrete workplace skills called observable practice activities (OPAs). Assessors rate behavior-based OPA tasks on a 5-level entrustment scale [29] In addition to OPA ratings, each assessment form has free text boxes for assessors to: 1) comment on areas of clinical strength, and 2) list two things this resident can do to improve [27, 29]. All assessment forms are stored in an electronic residency management system, MedHub (MedHub incorporated, Ann Arbor, MI). The University of Cincinnati institutional review board approved this study as exempt.

Given that many factors (e.g., rotation, time of year, assessor) can contribute to variation in observational assessment ratings,[30] we had previously created an expected entrustment score for every OPA using a linear mixed model with random effects that accounts for these factors. The difference between this expected score and the observed entrustment score (based on actual OPA ratings) is converted to a standard score (z-score) to estimate how far above (+) or below (−) a resident is from what would have been expected by the model. This z‑score, calculated for each month of residency training and cumulatively, serves as the reference standard for resident performance to distinguish between higher and lower entrusted residents. Dashboards to display these data are set to change color when a z-score is greater than one standard deviation below expected. We have been using this statistical modeling, a type of learning analytics, for several years to account for construct-irrelevant variance and help make sense of educational data [26, 31].

### Participants and data

When choosing our study population, we considered the average z‑score over the entire 36 months of residency for three consecutive cohorts of categorical internal medicine residents, who had entered the program in the 2014–15, 2015–16, and 2016–17 academic years. On average, each resident received 3738 subcompetency assessments (approximately 1246 OPA entrustment ratings) over 36 months of training. The average for the control population was 3795 and 3715 for the study population. We chose the cumulative z‑score to identify our study population because previous findings in our program of assessment have shown higher reliability and it lessened the risk of including residents with temporary struggles [26]. Twenty-six of these residents were greater than one standard deviation below the mean z‑score (38% female). These residents constituted our subjects of interest for this study and will henceforth be referred to as “lower entrusted” residents. As detailed below, we used a comparison group in the design of this study comprising “typically entrusted” residents, defined as all other residents not in the lower entrusted group. To ascertain this comparison group, a randomly generated sequence was used to select 13 residents with any z‑score above the lower entrusted group cutoff (46% female). Since the aim of our study was to find early themes that might predict future struggles, we collated all narrative data from the first 6 months of residency (July to December) for these 39 residents for analysis.

A program administrator entered narrative data for these residents into a Microsoft Excel (2019) spreadsheet and organized data by resident, month, and assessor type (faculty vs. peer and AHP). Two authors (JN and BD), not involved in the residency program or knowledgeable of any residents’ performance, assigned a number to each resident and blinded the entire spreadsheet by removing all references to names. We entered data into Dedoose (Los Angeles, CA: SocioCultural Research Consultants, LLC) to facilitate analysis.

### Analysis

We performed an inductive thematic analysis of the narrative data to ascertain two types of themes in these data: explicit/manifest, describing the literal or surface meaning (e.g., the specific weakness that assessors described) and implicit/latent, reflecting deeper meanings or assumptions (e.g., how assessors described weaknesses in their writing) [32]. The latter allowed for a richer description of the differences we identified in the data even when specific weaknesses were similar. To blind researchers from knowing which narrative comments came from lower compared with typically entrusted residents, we performed the analysis in two phases [33].

#### Phase 1

Initially, two authors (JN and BD) familiarized themselves with the data from both groups of residents compiled into one blinded data set by reading all narrative comments. Next, they independently analyzed narrative comments about five residents to create an initial codebook. After beginning with independent coding, they met to reach agreement. These authors then met with three additional authors (MK, BK, and DRS) to immerse themselves in the data, review initial codes, and develop consensus on both the codebook and application of codes to data from the first five residents. This process of two authors independently analyzing data and then coming to consensus with the three additional authors was repeated with data from another five residents followed by all remaining residents in the data set. The final coding was agreed upon by all authors involved in analysis to this point (JN, BD, MK, BK, DW, and DRS). These members of the author team then gathered codes into larger categories to group similar and related codes.

#### Phase 2

Two authors (JN and BD) unblinded all analyzed data to group the data into typical and lower entrusted resident categories to prepare for analyzing differences between the two groups. The two authors (JN and BD) then re-blinded the data and four members of the author team (MK, BK, DW, DRS), with extensive experience in assessment and reading narrative comments, independently analyzed a subset of the previously analyzed data between the typical and lower entrusted residents. This stage sought to further refine the previously defined categories to reflect similarities and differences between the two groups. Following individual analysis at this stage, these authors met to reconcile differences before meeting with the full author group on multiple occasions to confirm themes and finalize results.

## Results

We organized the narrative comments that differed between typical and lower entrusted residents into two types of themes: three explicit/manifest and three implicit/latent themes (Tab. 1). The three explicit/manifest themes focused on specific aspects of resident performance and are: 1) Gaps in attention to detail, 2) Communication deficits with patients, and 3) Difficulty recognizing the “big picture” in patient care for lower entrusted residents. The three implicit/latent themes focused on how narrative data were written and are: 1) Assessors describe feedback as a deficiency rather than an opportunity to improve for lower entrusted residents, 2) Assessors make normative comparisons that identified a resident as being behind their peers for lower entrusted residents, and 3) Assessors warn of possible risk to patient care for lower entrusted residents. Direct quotes are included and labelled with resident number, month, and assessor role.

**Table 1:** Themes present in the first six months of narrative data associated with lower overall entrustment at the end of an internal medicine residency

Six themes	Representative quotes
*Explicit/manifest: Resident performance*	
1. Gaps in attention to detail	*“There have been a few overlooks in regards to medications and orders that I have had to correct. I encouraged them to look at order and medication list on a daily basis as part of rounds to make sure that there is nothing important that is missing or needs to be removed”*
2. Communication deficits with patients	*“Bedside presentations include words that are not understandable to the patient”*
3. Difficulty recognizing the “big picture” in patient care	*“They could get a better handle of the overall picture of a patient instead of focusing only on the individual problems”*
*Implicit/latent: Assessor description*	
4. Describing feedback as a deficiency rather than an opportunity to improve	“*Their knowledge base overall is not good enough to answer simple questions such as how different insulins work etc.”*
5. Normative comparisons that identified a resident behind their peers	*“Knowledge base is below what would be expected for an early intern”*
6. Warning of possible risk to patient care	*“Supervising resident and attending need to keep close eye on them, look at everything”*

Before describing differences in narrative comments between groups, it is important to note that many similarities existed. We did not elaborate on these for the final analysis but note them here briefly for context. The most common similarity between groups focused on the need to further medical knowledge or knowledge acquisition, including generic advice to “read more” or specific areas for knowledge expansion. Other similarities included the need to broaden differential diagnoses, increase confidence in clinical practice, improve efficiency in documentation and workflow, and gain more clinical experience. We determined that analyzing comments that were similar between the groups would not contribute to our study aim, so we did not explore these themes further.

### Explicit/manifest theme #1: gaps in attention to detail

Comments describing a lack of attention to detail were common in the lower entrusted residents. These comments described a need for improved thoroughness and accuracy of completed tasks, such as knowing all the details of a patient’s current presentation and reviewing past medical history and previous admissions. Examples of faculty comments in this area included: *“needs to work on knowing the patient condition and collect[ing] and analyz[ing] the data more thoroughly”* (R7, Oct, Faculty) as well as the need to pay *“further attention to …chart review for new admissions [that allows a] better understanding of chronology of events in the recent past that inform the current admission” *(R18, Aug, Faculty).

Lower entrusted residents had comments about “*pay[ing] attention to detail when writing orders”* (R8, Dec, Faculty). They also had gaps in performing medication reconciliation on admission and discharge from the hospital, with one assessor noting “*more attention and analysis are needed on medication reconciliation*” (R15, Aug, Faculty).

Assessors frequently commented on the data acquisition skills of lower entrusted residents with comments such as “*be more thorough when obtaining a history from the patient” *(R32, Aug, Faculty). Lack of organization was sometimes noted in narrative comments as a possible explanation for lacking attention to details. This was sometimes accompanied by advice, such as the assessor who noted a resident should “*try making a check list of everything that needs done and cross off as you go” *(R32, Nov, AHP).

Comments about documentation tasks were also common, such as forgetting to update notes and sign-outs, and the need to remove inconsistent, repetitive, or inaccurate information. Illustrative faculty comments in this area include: *“notes suffer[ed] from copy/paste and are not thoroughly reviewed …every day and edit[ed] as appropriate.”* (R15, Nov, Faculty).

### Explicit/manifest theme #2: communication deficits with patients

Assessors identified communication deficits in lower entrusted residents. Often this took the form of suggestions to improve patient communication with three specific examples rarely found in typically entrusted residents. First, assessors suggested improving engagement with the patient through examples such as, *listening to patients, building rapport*, and *bedside manner*. Second, assessors suggested using less medical terminology that may be unclear to patients, such as *“bedside presentations include words *[that are]* not understandable to the patient” *(R29, Aug, Faculty). Finally, assessors suggested more clearly articulating a plan to the patient, avoiding “*a tendency to tell the patient too much”* (R28, Aug, Faculty) and tending to an inability to *“recognize when patients are not understanding what is being said.” *(R39, Nov, Faculty).

### Explicit/manifest theme #3: Difficulty recognizing the “big picture” in patient care

Many comments described an inability of lower entrusted residents to synthesize information and recognize the bigger picture in patient care. Assessors described residents getting “*bogged down with every detail” *and suggested* “keep*[ing]* an eye on the bigger picture” *(R6, Aug, Faculty). They described this being illustrated when a resident struggled to sort primary from secondary problems, resulting in “*difficulty prioritizing and then dealing efficiently with the most serious problems”* (R28, Sept, Faculty). Finally, assessors encouraged “*instead of focusing only on the individual problems …get a better handle of the overall picture *[of patient care]*” *(R12, Nov, Faculty).

### Implicit/latent theme #1: Assessors describe feedback as a deficiency rather than an opportunity to improve for lower entrusted residents

Assessors frequently used negative descriptors with lower entrusted residents compared with those with typical entrustment ratings, for whom constructive feedback was often framed as an opportunity to improve. Examples of negative descriptors from the former group included: “*disorganized*” and “… *poor self-confidence, which limits their capacity to propose a plan of care for the patients” *(R32, Dec, Faculty). In other cases, assessors explicitly used words such as “*deficiency,*” “*problem,*” “*concern,*” *“weakness,” “difficulty” *and “*struggle*” when narrative comments included constructive feedback. Illustrative narrative comments employing these terms include: “*struggled developing a system of organization” *(R31, Sept, Faculty), *“… concern about the level of detail for their progress notes”* (R2, Nov, Faculty), and “*difficulty synthesizing information *[for basic tasks]*” *(R13, Aug, Faculty).

Another way that assessors expressed negative narrative comments with lower entrusted residents was to call direct attention to something the assessor felt should have been done but was not by using the phrase “*did not.*” Examples include: “*they did not present or possibly even find history of mitral valve repair” *(R2, Dec, Faculty)* or “did not come up with a differential diagnosis” *(R37, Sept, AHP) *or “they did not report her ‘white out chest x‑ray’” *(R2, Dec, Faculty).

Finally, demonstrating the most extreme limit of this theme, assessors sometimes clearly conveyed a value-laden negative tone, such as describing a resident as *“oblivious to what was going on”* (R7, July, AHP) or “*this intern glosses over things they do not understand”* (R26, Dec, Faculty). This was also reflected in describing opportunities for improvement as concern for the resident’s potential ability to perform better. This is illustrated well by a faculty assessor who noted a resident’s *“knowledge base overall is not good enough to answer simple questions such as how different insulins work.”* (R26, Sept, Faculty).

### Implicit/latent theme #2: Assessors make normative comparisons that identified lower entrusted residents as behind their peers

When documenting narrative comments for lower entrusted residents, assessors sometimes used normative language. For example, faculty noted that a resident is “*not at the same level as co-interns” *(R37, Sept, AHP), “*below what would be expected for an early intern” *(R35 Sept, Faculty), and “*lack*[ing]* more self-confidence than others …” *(R26, Dec, Faculty). In other examples, assessors used less obvious examples while invoking that a resident needed more help than their peers with comments such as, *“relied heavily on senior to incorporate* [information]* independently”* (R25, Oct, AHP) or a comment that a resident was *“in the early stages”* (R18 Dec, Faculty) for many basic tasks.

### Implicit/latent theme #3: Assessors warn of possible risk to patient care for lower entrusted residents

Assessors used more language signaling risk to patients in two ways. First, concern over risk to patients was documented through using terms that describe potentially unsafe care, such as “*mistake,*” “*inaccurate,*” or “*errors.*” Examples include: *“sometimes errors were caught in discharge med recs”* (R21, Nov, AHP) and *“even with direct supervision by a senior resident* [presentations and examination skills] *were often inaccurate.” *(R25, Oct, AHP) Second, risk concerns were described by conveying feelings about the potential for errors, including calls for closer supervision, using words such as “*worry*” and “*concern*” or even directly stating *“supervising resident and attending need to keep close eye on them, look at everything” *(R2, Dec, Faculty).

## Discussion

We identified themes in narrative comments during the first 6 months of training that were present in residents who subsequently had lower entrustment ratings during residency, dividing themes into explicit/manifest and implicit/latent to explore differences in both residents’ performance and how assessors describe that performance. Narrative data can differ between higher and lower performing residents and thus can be used to discriminate between learners [18, 24]. Many faculty members describe their reading of narrative data as scanning for red flags, usually in the form of words or phrases [23]. We advanced this understanding of red flags by exploring themes in the narrative comments that were unique to learners who subsequently had lower entrustment ratings. While data suggesting extreme outlier performance can usually help identify residents with performance concerns, CCCs often rely on accumulated data and trends, both of which take time and potentially delay early identification [34]. Our findings could aid CCCs in their incorporation of narrative comments to support specific residents that may benefit most from earlier intervention. We hope these findings continue building emphasis on the implications of how narrative data can be used to guide decision-making (including predictive analytics and machine learning algorithms) in a program of assessment [20, 35].

In programs of assessment, numerical and narrative data are often obtained for formative purposes but used by CCCs to make summative decisions about a learner’s trajectory [36, 37]. Recognizing the right time to intervene on perceived concerns can be complicated. CCCs face two challenging scenarios: *overreacting* to specific comments and implementing remediation when it may not be necessary or *underreacting* and not intervening while waiting for more data despite valuable time passing to help a struggling resident. Differentiating signal from noise is a challenge in all early assessment efforts, although evidence suggests minimal narrative data is needed to discriminate between learners [18]. Our findings can be helpful in determining when to intervene and when to simply continue monitoring, allocating resources (faculty time, extra CCC discussion, remediation plans, etc.) where there is a higher likelihood of learners having continued struggles. Specifically, comments invoking the need to increase knowledge, build confidence, gain experience, and improve efficiency in workflow or documentation were present in both typical and lower entrusted residents. Therefore, these types of comments are less likely to help identify residents early in training who need additional intervention. However, comments describing a lack of attention to detail, difficulty communicating clearly with patients, or synthesizing details to see a bigger picture are potentially more likely to portend ongoing struggles, prompting a swifter reaction to consider whether intervention is warranted.

We found that beyond the specific details of performance, sometimes specific descriptors can also be a signal in lower entrusted residents. The adage, “it’s not what you say, but how you say it” applies to both verbal and written narrative feedback. Recent studies on narrative data support that faculty have consistent writing styles and uncovering meaning often requires reading beyond the literal words [21–24, 38]. We found certain implicit/latent themes in written narrative feedback were disproportionately present in lower entrusted residents. These themes represent another layer in CCCs decision-making as they encounter narrative data that describe a resident as behind their peers, warning of risk to patient safety, and framing their feedback as a deficiency. When narrative feedback explores common themes for all early residents (i.e. knowledge, efficiency, confidence) the implicit/latent characteristics represent an opportunity to still uncover signal in the noise.

Finally, in addition to harnessing and building upon our findings to identify signal, the process of using an iterative qualitative lens to analyze narrative data in a large program of assessment is transferable to other programs. Narrative data are often difficult to interpret for individuals, but more easily understood when viewed in aggregate [22]. Viewing narrative data in cohorts with larger aggregates, as seen in this study, can yield additional insights. Since meaning is contextual and dependent on cultural norms, programs analyzing their own narrative data for keywords and patterns may provide deeper understanding of comments that require more immediate and definitive interventions [21]. This can aid CCCs in better recognition of patterns or inform text-based applications of machine learning algorithms, to help predict those residents that might benefit most from limited resources and earlier intervention to improve their developmental trajectory [35].

### Limitations

First, we analyzed data from one internal medicine residency program, which may limit transferability of the findings to other programs. Specifically, these findings may be more specific to medical-based training programs and less applicable to procedure-based specialties. Second, we used learning analytics to define typical and lower performance using quantitative ratings. However, those ratings as well as our modeling may not accurately categorize trainees by their performance. This possibility noted, we believe our program of assessment as well as performance analytic modeling are robust. Third, a sample of typically performing residents was analyzed and it is possible that if larger samples had been used the contrast between themes in lower performing residents could have changed. Fourth, given our methodology we cannot assert that the themes in the narrative data will predict residents who will struggle. Finally, we did not compare or contrast comments from different assessors and therefore we do not know if assessor-specific characteristics might impact the type or description of narrative data provided. Future study should explore this.

## Conclusion

Using the themes in this study as a lens to review narrative comments may help CCCs with earlier recognition and interventions to support residents’ development. Future studies should continue to investigate the implications of using narrative data to guide decision-making and predict those that will struggle most.
